# lncRNA–mRNA Expression Patterns in Invasive Pituitary Adenomas: A Microarray Analysis

**DOI:** 10.1155/2022/1380485

**Published:** 2022-05-05

**Authors:** Chao Peng, Shuaikai Wang, Jinxiu Yu, Xiaoyi Deng, Huiyu Ye, Zhishan Chen, Hongru Yao, Hanjia Cai, Yanli Li, Yong Yuan

**Affiliations:** ^1^Department of Neurosurgery, Guangdong Provincial People's Hospital, Guangdong Academy of Medical Sciences, Guangzhou, Guangdong 510080, China; ^2^Department of Neurosurgery, Shenzhen Luohu People's Hospital, Shenzhen, Guangdong 518001, China; ^3^Department of Radiotherapy, The Second Affiliated Hospital of Guangzhou Medical University, Guangzhou, Guangdong 510260, China; ^4^Department of Endocrinology, The Second Affiliated Hospital of Guangzhou Medical University, Guangzhou, Guangdong 510260, China; ^5^Guangzhou Medical University, Guangzhou, Guangdong 510000, China; ^6^Department of Neurosurgery, The Second Affiliated Hospital of Kunming Medical University, Kunming, Yunnan 650101, China

## Abstract

**Background:**

Long noncoding RNAs (lncRNAs) play important roles in the tumorigenesis and progression of various cancer types; however, their roles in the development of invasive pituitary adenomas (PAs) remain to be investigated.

**Methods:**

lncRNA microarray analysis was performed for three invasive and three noninvasive PAs. Gene Ontology (GO) enrichment and Kyoto Encyclopedia of Genes and Genomes (KEGG) analysis were performed, and coexpression networks between lncRNA and mRNA were constructed. Furthermore, three differentially expressed lncRNAs were selected for validation in PA samples by real-time quantitative reverse transcription polymerase chain reaction (qRT-PCR). The diagnostic values of these three lncRNAs were further evaluated by a receiver operating characteristic (ROC) curve analysis.

**Results:**

A total of 8872 lncRNAs were identified in invasive and paired noninvasive PAs via lncRNA microarray analysis. Among these, the differentially expressed lncRNAs included 81 that were upregulated and 165 that were downregulated. GO enrichment and KEGG pathway analysis showed that these differentially expressed lncRNAs were associated with the posttranslational modifications of proteins. Furthermore, we performed target gene prediction and coexpression analysis. The interrelationships between the significantly differentially expressed lncRNAs and mRNAs were identified. Additionally, three differentially expressed lncRNAs were selected for validation in 41 PA samples by qRT-PCR. The expression levels of FAM182B, LOC105371531, and LOC105375785 were significantly lower in the invasive PAs than in the noninvasive PAs (*P* < 0.05). These results were consistent with the microarray data. ROC curve analysis suggested that the expression levels of FAM182B and LOC105375785 could be used to distinguish invasive PAs from noninvasive PAs.

**Conclusion:**

Our findings demonstrated the expression patterns of lncRNAs in invasive PAs. FAM182B and LOC105375785 may be involved in the invasiveness of PAs and serve as new candidate biomarkers for the diagnosis of invasive PAs.

## 1. Background

Pituitary adenomas (PAs), one of the most common intracranial tumors, account for 10%–20% of intracranial tumors [1]. According to tumor biological characteristics, PAs can be divided into noninvasive PAs (NIPAs), invasive PAs (IPAs), and pituitary adenocarcinoma [2]. IPAs, which are highly proliferative and invasive, tend to invade vital surrounding structures, such as the cavernous sinus, sphenoid bone, and cranial nerves [3]. Owing to the aggressive behavior of IPAs, the cure rate of complete removal by surgical resection is low, whereas the incidence of recurrence is high. Therefore, the identification of novel biomarkers for early diagnosis that reflect the clinicopathological behaviors of IPAs is important. Additionally, the exploration of molecular mechanisms involved in the invasiveness of PAs is urgently needed.

lncRNAs, which are >200 nucleotides in length, are involved in various processes of gene regulation, such as nuclear and cytoplasmic trafficking, chromosome dosage compensation, and mRNA splicing and translation [4]. With the development of high-throughput sequencing technologies, accumulating evidence has indicated that the expression of lncRNA is associated with various tumors [5, 6]. Furthermore, lncRNAs have been increasingly identified as novel diagnostic and prognostic markers of various tumors [7, 8]; however, the roles of lncRNAs in PAs remain to be further investigated.

Xue and Ge [9] identified differentially expressed lncRNAs in PAs and revealed the key lncRNAs associated with the progression of PAs. Moreover, Guo et al. [10] showed that some lncRNAs were associated with the recurrence of nonfunctioning PAs, whereas Zhu et al. [11] demonstrated that the increased expression levels of the lncRNA maternally expressed 8 promoted bone destruction in bone-invasive PAs by regulating miR-454-3p/tumor necrosis factor (TNF)-*α* axis. Thus, exploring the expression patterns of lncRNAs in IPAs might confirm the existence of novel potential biomarkers for the diagnosis of IPAs.

This study aimed to investigate the expression patterns of lncRNAs in both IPAs and NIPAs. We performed microarray analysis to reveal the expression profiles of lncRNAs in three IPAs and three NIPAs. Subsequently, we identified the differentially expressed lncRNAs in IPAs and constructed lncRNA–mRNA networks. Finally, among the differentially expressed lncRNAs, three lncRNAs were selectively examined by real-time quantitative reverse transcription polymerase chain reaction (qRT-PCR) in a large sample size for further validation. These were later used for the diagnosis of IPAs. These findings could provide novel insights on the mechanisms underlying the invasive behaviors of PAs. Accordingly, lncRNAs may even be novel biomarkers for the diagnosis of IPAs.

## 2. Materials and Methods

### 2.1. Patients and Samples

**Figure 1:**
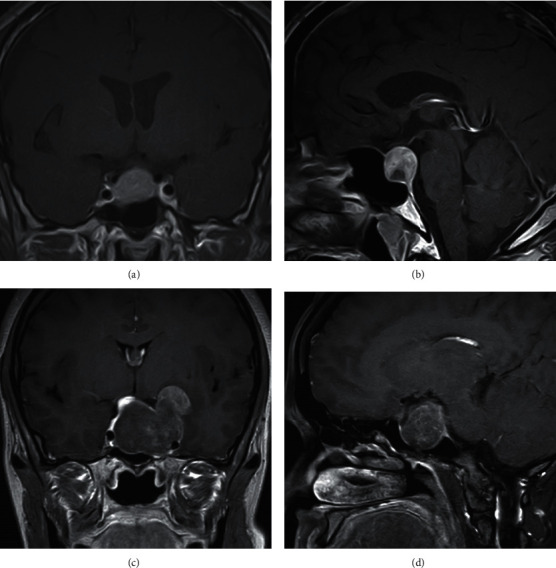
Enhanced magnetic resonance imaging of two patients with noninvasive pituitary adenoma (NIPA) and invasive pituitary adenoma (IPA). (a, b) Coronal and sagittal scans of the NIPA, respectively. (c, d) Coronal and sagittal scans of the IPA invading the left cavernous sinus and surrounding the internal carotid artery (Knosp classification grade IV).

Tumor specimens were obtained from patients with PAs who underwent transsphenoidal surgery at the Department of Neurosurgery of the Guangdong Provincial People's Hospital (Guangzhou, China) between January 2020 and June 2021. The diagnosis of PA was based on clinical manifestations, biochemical features of hormonal secretion, magnetic resonance imaging (MRI), and histopathological analysis confirmed by two pathologists after surgical resection. NIPA was defined as the limitation of the tumor mass within the sellar region, without any compression on peripheral structures (Figures 1(a) and 1(b)). IPA was defined according to Knosp classification grades III–IV [12] (Figures 1(c) and 1(d)). IPAs (*n* = 3) and NIPAs (*n* = 3) were selected for lncRNA microarray analysis. The details of these six PAs are shown in Table 1. In addition, another 41 specimens of PAs, including IPAs (*n* = 21) and NIPAs (*n* = 16), were used for qRT-PCR validation. The clinical characteristics of the 41 patients with PAs are summarized in Table 2. Tumor dimensions were manually obtained from MRI. A microadenoma was defined by a maximal tumor diameter of <10 mm, a macroadenoma was ≥10 mm, a large macroadenoma was ≥20 mm, and a giant adenoma was ≥40 mm. The dimensional indices of the tumors were measured and recorded in three orthogonal planes: transverse (TR), anteroposterior (AP), and craniocaudal (CC). The tumor volumes were estimated using the following formula: V = (*π* × [TR × AP × CC])/6 [13]. After surgical resection, all tissue samples were immediately frozen in liquid nitrogen and stored at −80°C for further analyses. All procedures used in this study were approved by the Ethics Committee of Guangdong Provincial People's Hospital. Informed consent was obtained from all patients.

### 2.2. Total RNA Extraction and Purification

Total RNA was extracted using TRIzol reagent (Invitrogen, Carlsbad, USA) according to the manufacturer's protocol. Furthermore, total RNA was quantified using Bioanalyzer 2200 (Agilent, California, USA) and kept at −80°C. The RNA samples with RNA integrity number of >6.0 were acceptable for rRNA depletion and subsequent lncRNA purification. The purification of total RNA was validated by gel electrophoresis.

### 2.3. cDNA Library Construction

cDNA libraries were constructed for each pooled RNA sample using the NEBNext® Ultra™ Directional RNA Library Prep Kit (New England BioLabs Inc., MA, USA) for Illumina according to the manufacturer's instructions. The protocol comprised the following steps: mRNA molecules were fragmented into 150–200 bp using divalent cations at 94°C for 8 min. The cleaved RNA fragments were used as templates and reverse-transcribed into first-strand cDNA. Subsequently, the second-strand cDNA was synthesized using polymerase I and RNase H with the reaction buffer. Target bands were harvested using AMPure XP Beads (Beckman Coulter, CA, USA). The products were then purified and enriched by PCR to create the final cDNA libraries and quantified using Bioanalyzer 2200 (Agilent). The tagged cDNA libraries were pooled in equal ratio and used for 150-bp paired-end sequencing in a single lane of Illumina HiSeq XTen. cDNA library construction and RNA sequencing were completed at the Center of NovelBio Lab (Shanghai, China).

### 2.4. Mapping and Identification of Differentially Expressed Genes

Before read mapping, clean reads were obtained from the raw reads by removing the adaptor sequences, reads with >5% ambiguous bases (noted as N), and low-quality reads containing >20% bases with qualities of <20. The clean reads were then aligned to the human genome (version: GRCh38 NCBI) using Hierarchical Indexing for Spliced Alignment of Transcripts v2.1.0 (HISAT2) [14]. HTSeq was used to count genes and lncRNAs, whereas the reads per kilobase per million mapped reads method was used to determine gene expression [15]. We used the DESeq algorithm [16] to filter the differentially expressed genes after analyzing the level of significance, i.e., the determination of *P* value, and false discovery rate (FDR) under the following criteria: (1) fold change of >2 or<0.5 and (2) FDR of <0.05 [17].

### 2.5. Functional Enrichment Analysis

Gene Ontology (GO) enrichment was performed to elucidate the biological implications of differentially expressed genes [18]. We downloaded the GO annotations from NCBI (http://www.ncbi.nlm.nih.gov/), UniProt (http://www.uniprot.org/), and GO (http://www.geneontology.org/) databases. Fisher's exact test was performed to identify the significant GO categories, and FDR was used to correct the *P* values. GO is structured as a directed acyclic graph, and each term has defined relationships with one or more other terms. A GO-Tree was built based on the GO directed acyclic graph to provide user-friendly data navigation and visualization. We selected the significant GO terms (*P* < 0.01) obtained from GO enrichment based on the differentially expressed genes to construct the GO-Tree for summarizing the affected functions [19].

Kyoto Encyclopedia of Genes and Genomes (KEGG) analysis was performed to clarify the roles and significance of target genes in the overall biological pathways [20]. We selected the genes in enriched biological pathways and used Cytoscape to obtain graphical representations of the pathways [21]. The KEGG database was used to build the network of genes according to the relationship among the genes, proteins, and compounds in the database.

### 2.6. Construction of the lncRNA–miRNA–Target Gene Interaction Network

The role of lncRNAs in IPAs was investigated based on an lncRNA–miRNA–target gene interaction network. According to the results of lncRNA microarray analysis, the 10 most dysregulated lncRNAs in IPAs were selected and Cytoscape was used to map out an interaction network. Putative interactions between lncRNAs and miRNAs were predicted using two online databases: Jefferson Computational Medicine Center-RNA22 v2 microRNA target detection (https://cm.jefferson.edu/rna22/Interactive/) and LncBase Predicted v.2 (http://carolina.imis.athena-innovation.gr/diana_tools/web/index.php?r=lncbasev2%2Findex-predicted). Subsequently, miRNAs with the highest target scores were selected and their target genes were evaluated using TargetScan [22] and miRanda [23]. Finally, miRNAs and their target genes with high targeting-relationship scores were selected to construct the lncRNA–miRNA–target gene interaction network. The interaction network was delineated using Cytoscape.

### 2.7. qRT-PCR Validation

Total RNA from 41 specimens of PAs was extracted using TRIzol reagent (Invitrogen). Reverse transcription and qRT-PCR were performed using a Reverse Transcription Kit (Takara, Dalian, China) and PrimeScript RT Reagent Kit (Takara, Dalian, China), respectively, as previously described [24]. The expression of lncRNAs was measured by qRT-PCR. The sequences of the primers used are listed in Table 3. The gene expression levels were normalized to *actin*. They were determined by the 2^−*ΔΔ*Ct^ method and analyzed for statistical significance.

### 2.8. Statistical Analysis

Measurement data are presented as mean ± standard error of the mean (SEM) and enumeration data are presented as percentages. Comparisons were performed using independent samples t-test between pairs of groups or one-way analysis of variance for more than two groups followed by Dunnett's multiple comparison test. The receiver operating characteristic (ROC) curves and the area under the curve (AUC) were used to estimate the diagnostic power and accuracy of lncRNAs in IPAs and NIPAs. All statistical analyses were performed using Statistical Product and Service Solutions (SPSS) 25.0 software (SPSS Inc., Chicago, IL, USA). *P* value of <0.05 was considered statistically significant, which is indicated in the figures.

## 3. Results

### 3.1. Identification of Differentially Expressed lncRNAs between IPAs and NIPAs

**Figure 2:**
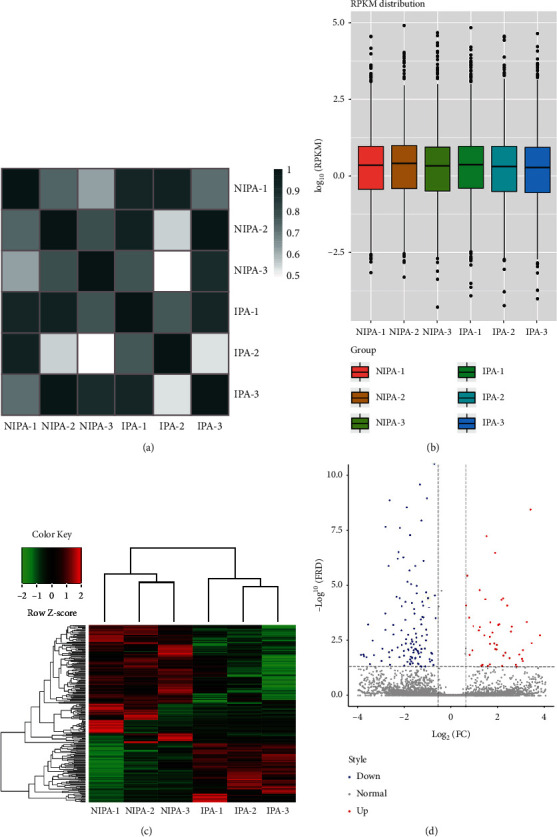
Long noncoding RNA (lncRNA) profiles obtained by microarray analysis. (a) Correlation among the six samples based on the expression values of the differentially expressed lncRNAs. (b) Boxplots of intensity values obtained by microarray analysis. (c) Heat map based on the expression values of significantly differentially expressed lncRNAs with fold change of >2 or <0.5 and *P* value of <0.05. Red and green indicate increased and decreased expression levels, respectively. (d) Volcano plot showing differentially expressed lncRNAs between invasive and noninvasive pituitary adenomas, with red dots indicating upregulation and green dots indicating downregulation.

To identify the differentially expressed lncRNAs in IPAs and NIPAs, we performed high-throughput human lncRNA microarray analysis using three IPAs and three NIPAs. The correlation plot was used to detect the correlation between microarray samples and confirm the homogeneity between biological replicates (Figure 2(a)). The box plot demonstrated that the distributions of normalized intensities were almost identical among all samples (Figure 2(b)). Furthermore, hierarchical clustering was used to illustrate the significantly differentially expressed lncRNAs in the two groups (Figure 2(c)). Volcano plots were used to reveal the variations in the expression levels of lncRNAs between the two groups (Figure 2(d)). Overall, 8872 lncRNAs and 16039 mRNAs were identified in PAs via human lncRNA microarray analysis. Among these, 246 lncRNAs were differentially expressed in IPAs compared with NIPAs, including 81 upregulated and 165 downregulated lncRNAs (Figure 2(d)). Furthermore, 566 mRNAs were differentially expressed in IPAs compared with NIPAs, including 289 upregulated and 277 downregulated mRNAs. These results suggested that the expression of lncRNAs in IPAs was different from that in matched NIPAs.

### 3.2. Delineation of GO Enrichment and KEGG Pathway Analysis

**Figure 3:**
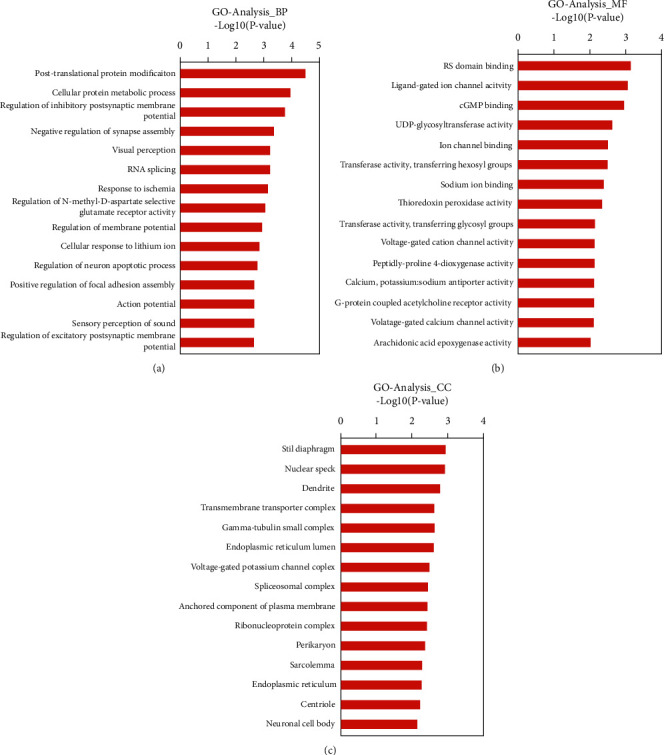
Gene ontology (GO) terms from the biological process (BP) (a), molecular function (MF) (b), and cellular component (CC) (c). Functional analysis of differentially expressed lncRNAs and coexpressed mRNAs for GO enrichment.

To further investigate the functional roles of the differentially expressed lncRNAs, GO enrichment and KEGG pathway analysis were performed. GO terms were classified into three different domains: biological processes (BPs), molecular functions (MFs), and cellular components (CCs). The top 15 generally changed GO terms were ranked by fold enrichment or enrichment score as listed in Figure 3. The top five identified BPs were posttranslational protein modification, cellular protein metabolic process, regulation of inhibitory postsynaptic membrane, negative regulation of synapse assembly, and visual perception (Figure 3(a)). The top five identified MFs were RS domain binding, ligand-gated ion channel activity, cyclic guanosine monophosphate binding, uridine diphosphate–glycosyltransferase activity, and ion channel binding (Figure 3(b)). Furthermore, the top five identified CCs were slit diaphragm, nuclear speck, dendrite, transmembrane transporter complex, and gamma-tubulin small complex (Figure 3(c)).

**Figure 4:**
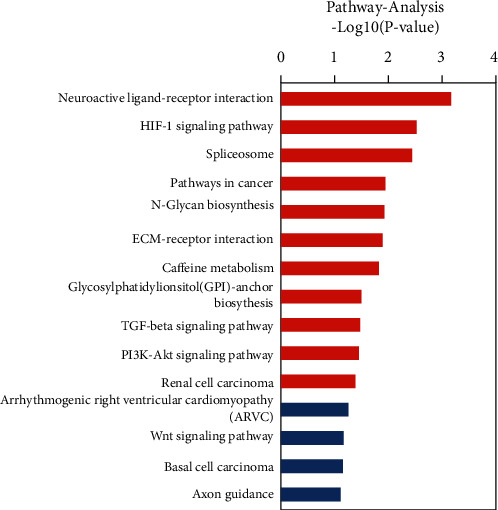
Kyoto Encyclopedia of Genes and Genomes (KEGG) pathway analysis of differentially expressed long noncoding RNAs (lncRNAs) and coexpressed mRNAs. The length of the column indicates the *P* value. The longer the column and the lower the *P* value, the more enriched and meaningful the pathway.

The KEGG database was used to identify the pathways and molecular interactions associated with the target genes. Our data indicated that the target genes were mostly enriched in neuroactive ligand–receptor interaction, hypoxia-inducible factor 1 alpha (HIF-1) signaling pathway, spliceosome, cancer pathways, and N-glycan biosynthesis (Figure 4). According to these results, these pathways may contribute significantly to the invasiveness of PAs.

### 3.3. Construction of the lncRNA–miRNA–mRNA Coexpression Network

**Figure 5:**
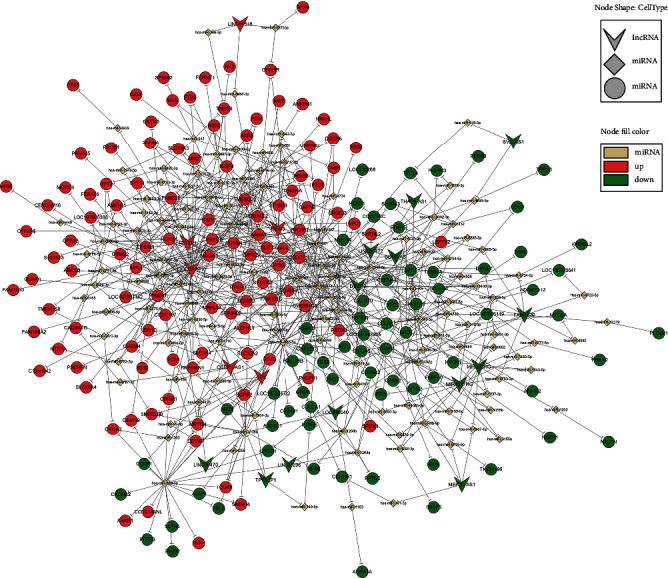
Interaction network of long noncoding RNAs (lncRNAs)–miRNAs–target genes. The arrows, rhombuses, and circles indicate lncRNAs, miRNAs, and mRNAs, respectively. Red and blue colors signify upregulation and downregulation, respectively.

To reveal the potential functions and mechanisms of differentially expressed lncRNAs in IPAs, an lncRNA–miRNA–mRNA coexpression network was constructed based on bioinformatics analysis. The most down- and upregulated lncRNAs in IPAs were selected for constructing the lncRNA–miRNA–mRNA coexpression network, which was delineated using Cytoscape (Figure 5).

### 3.4. Validation of Differentially Expressed lncRNAs in PAs by qRT-PCR

**Figure 6:**
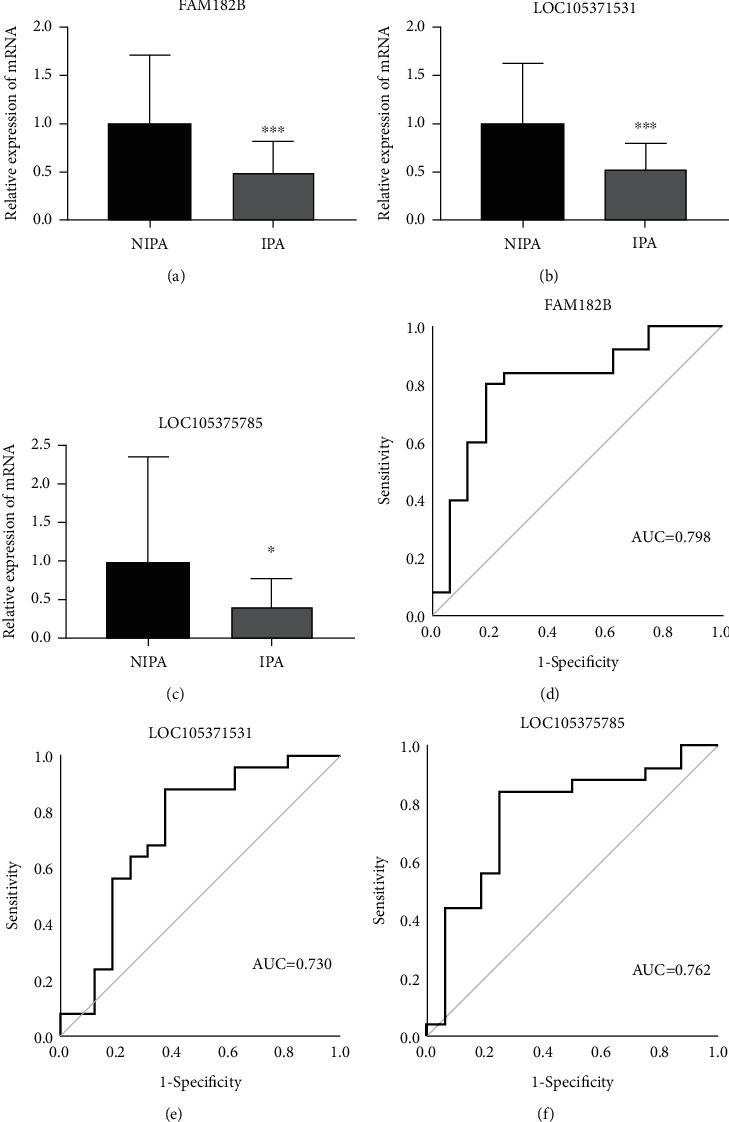
Validation of the results of long noncoding RNA (lncRNA) microarray analysis by real-time quantitative reverse transcription polymerase chain reaction (qRT-PCR) and receiver operating characteristic (ROC) curve analysis for the three selected lncRNAs. (a–c) Expression levels of the three selected lncRNAs—FAM182B, LOC105371531, and LOC105375785—in invasive pituitary adenomas (IPAs) (*n* = 25) and noninvasive pituitary adenomas (NIPAs) (*n* = 16). (d–f) ROC curves of the three selected lncRNAs between IPAs and NIPAs. Data is represented as mean ± SEM, ∗*P* < 0.05, ∗∗*P* < 0.01, and ∗∗∗*P* < 0.001 versus NIPAs.

To validate the results of microarray analysis, three significantly differentially expressed lncRNAs—FAM182B, LOC105371531, and LOC105375785—were selected for validation by qRT-PCR in 25 IPAs and 16 NIPAs. The results of qRT-PCR confirmed that the expression of all three lncRNAs was significantly decreased in IPAs than in NIPAs (*P* < 0.05) (Figures 6(a)–6(c)). The results of qRT-PCR were consistent with those of microarray analysis, confirming the high reliability of the microarray analysis data.

### 3.5. Diagnostic Value of the Three Selected lncRNAs for IPAs

ROC curve analysis was performed to determine the diagnostic sensitivity and specificity of the three selected lncRNAs for IPAs (Figures 6(d)–6(f)). The AUCs for FAM182B, LOC105371531, and LOC105375785 were 0.798 [95% confidence interval (95% CI): 0.650–0.945], 0.730 (95% CI: 0.557–0.945), and 0.762 (95% CI: 0.604–0.921), respectively. These data suggested that FAM182B and LOC105375785 can be used to distinguish patients with IPAs from those with NIPAs if an AUC of ≥0.75 is considered diagnostically significant for the biomarker.

### 3.6. Correlation between the Expression of FAM182B and the Clinical Features of Patients with PAs

Considering that the highest AUC value was obtained for FAM182B, we conducted further investigation on this lncRNA. Using the median values of FAM182B expression levels in all patients as the boundary, we further divided the 41 patients with PAs into high and low FAM182B expression subgroups. The analysis demonstrated that FAM182B expression was not associated with sex, age, Ki-67 percentage, tumor volume, and surgical extent. However, a significant relationship was found between invasive behavior and FAM182B expression (*P* = 0.001) (Table 4). Further, FAM182B expression was significantly higher in patients with IPAs than in patients with NIPAs (*P* = 0.001) (Table 4).

## 4. Discussion

lncRNAs, a class of RNA transcripts longer than 200 nucleotides, can regulate the expression of protein-coding genes at the transcriptional and translational levels [25]. lncRNAs are involved in various processes related to gene regulation [26]. Accumulating evidence has indicated that the expression of lncRNAs is associated with various tumors and can be a promising biomarker for the diagnosis of tumors [27]. PAs, one of the most common intracranial tumors, may invade important surrounding structures, including the cranial bone and sphenoid bone [3]. The mechanisms associated with the invasion of PAs and novel biomarkers for the diagnosis of IPAs remain largely unclear. Recently, several lncRNAs, including small nucleolar RNA host gene 1 (SNHG1), H19, colon cancer associated transcript 2, LINC00473, and antisense noncoding RNA in the INK4 locus, were reported to participate in the proliferation, progression, and invasion of PAs [28–31]. However, the lncRNA–mRNA expression patterns and dysregulated lncRNAs in IPAs remained to be investigated.

In this study, lncRNA microarray analysis was performed to investigate the lncRNA expression patterns in IPAs and NIPAs. We identified 246 lncRNAs that were significantly differentially expressed, 81 of which were upregulated and 165 were downregulated in IPAs. Subsequently, these differentially expressed lncRNAs were integrated into hierarchical categories according to the heat maps. We observed that the lncRNA expression patterns were remarkably different between IPAs and NIPAs. The results indicated that lncRNAs are involved in regulating the invasiveness of PAs. These results are consistent with the findings of previous studies, which reported that lncRNAs are involved in the invasive behaviors of tumors [32, 33]. However, the results of our expression pattern and pathway analyses were different from those of a previous study conducted using the GSE26966 database [9]. This difference may be because pituitary gonadotrope tumors were used for the microarray analysis in the GSE26966 database.

To further identify the potential function of these differentially expressed lncRNAs, GO enrichment and KEGG pathway analysis were performed. Notably, the most significant GO terms for the differentially expressed lncRNAs were related to posttranslational protein modifications, which were previously reported to be important in the development of PAs [34]. KEGG pathway analysis of the differentially expressed lncRNAs revealed that neuroactive ligand–receptor interaction and HIF-1 signaling pathway may serve pivotal roles in the invasive mechanisms of PAs as they were more likely to be identified in IPAs than in NIPAs. Previous studies demonstrated that the expression levels of HIF-1 were significantly higher in IPAs than in NIPAs and that the HIF-1 signaling pathway promoted the invasiveness of PAs [35, 36]. Hou et al. [37] reported that differentially expressed genes in pituitary gonadotroph adenomas had enriched neuroactive ligand–receptor interaction pathways, which is consistent with our results.

Further analysis of three dysregulated lncRNAs from the tissues of 25 IPAs and 16 NIPAs confirmed the reliability of the results of lncRNA microarray analysis. Additionally, the three validated lncRNAs—FAM182B, LOC105371531, and LOC105375785—were downregulated in IPAs and could be used to distinguish IPAs from NIPAs. These results collectively demonstrated that lncRNAs may be implicated in the invasive behaviors of PAs. Accumulating evidence has also shown that lncRNAs can be promising biomarkers for various cancers [38, 39]. For instance, Liu et al. [40] demonstrated that SNHG16 can be a potential biomarker for hepatocellular carcinoma, whereas Teng et al. [41] showed that lung cancer associated transcript 1 acts as a potential biomarker for gastric cancer. In our study, both FAM182B and LOC105375785 showed relatively high specificity and sufficient sensitivity for the diagnosis of IPAs by ROC curve analysis. These results collectively demonstrated that lncRNAs may function as promising novel biomarkers for the diagnosis of IPAs. Moreover, FAM182B has been reported to be associated with hepatocellular carcinoma [42]. In our study, FAM182B, which had the highest AUC value among the three validated lncRNAs, was significantly associated with the invasive behavior of PAs.

Notably, there are three main limitations of this study. First, we did not perform the functional confirmation of these differentially expressed lncRNAs to clarify the functions and mechanisms of lncRNAs in the invasiveness of PAs. Second, the number of PAs was relatively small, which may limit the statistical power. The possible clinical implications of lncRNAs in the diagnosis of IPAs remain to be elucidated using a larger number of samples from patients with IPAs. Third, considering the irregular shape of the tumors, the tumor volume measurements were only a rough estimate of the actual tumor volumes in this study.

In conclusion, our results revealed the expression profiles of differentially expressed lncRNAs in IPAs using microarray analysis. Furthermore, GO enrichment and KEGG pathway analysis were performed to identify the potential functions of the differentially expressed lncRNAs. Additionally, lncRNA–mRNA coexpression networks were constructed. Taken together, three validated lncRNAs—FAM182B, LOC105371531, and LOC105375785—may be promising biomarkers for differentiating IPAs from NIPAs. Nevertheless, further studies are warranted to elucidate the detailed functions and mechanisms of lncRNAs in the invasive behaviors of PAs.

## Figures and Tables

**Table 1:** Details of three IPAs and three NIPAs used for microarray analysis.

ID	Sex	Age (years)	Tumor size (mm)	Secretory function	Knosp grade
IPA-1	Male	21	25∗20∗38	Nonfunctioning	IV
IPA-2	Male	50	24∗26∗18	Nonfunctioning	III
IPA-3	Female	57	23∗22∗18	Nonfunctioning	IV
NIPA-1	Male	41	35∗24∗25	Nonfunctioning	I
NIPA-2	Male	56	29∗26∗19	Nonfunctioning	I
NIPA-3	Female	38	14∗18∗18	Nonfunctioning	II

**Table 2:** Characteristics of 41 patients with PAs.

Characteristic	NIPA	IPA
*N*	16	25
Age (years)	43.56 ± 10.46	46.08 ± 14.04
Male, *n* (%)	11 (68.8)	14 (56.0)
Secretory function, *n* (%)		
Nonfunctioning	9 (56.2)	16 (64.0)
PRL	1 (6.3)	5 (20.0)
GH	4 (25.0)	2 (8.0)
ACTH	2 (12.5)	1 (4.0)
TSH	0	1 (4.0)
Median tumor volume (cm^3^)	4.60 (0.18-22.05)	4.61 (1.41-11.08)
Tumor size, *n* (%)		
Microadenoma	0	0
Macroadenoma	5 (31.3)	1 (4.0)
Large macroadenoma	9 (56.2)	20 (80.0)
Giant adenoma	2 (12.5)	4 (16.0)
Surgical extent, *n* (%)		
Gross total resection	14 (87.5)	17 (68.0)
Residual	2 (12.5)	8 (32.0)

PRL: prolactin hormone; GH: growth hormone; ACTH: adrenocorticotropic hormone; TSH: thyroid-stimulating hormone.

**Table 3:** Oligonucleotide sequences of primer for qRT-PCR analysis.

Gene symbol	Forward primer	Reverse primer
Actin	TGTGGATCGGTGGCTCCATCCT	AAACGCAGCTCAGTAACAGTCCGC
FAM182B	GCACTCTGGGTCCTGTTCTC	CACTTCCCTGCCTCCTACAC
LOC105371531	CAGGGTTATGAGATCGTC	GTTTCTGGGTCTTGGAGT
LOC105375785	ATCATCACTCTGCCCACCAT	AGTCGGATGACCTCCTCCTT

**Table 4:** Association between FAM182B expression and clinical characteristics.

Variable	FAM182B expression		Univariate analysis	
	Low (*n* = 20)	High (*n* = 21)	*χ* ^2^	*P* value
Gender				
Male	11 (55.0%)	14(66.7%)	0.586	0.444
Female	9 (45.0%)	7 (33.3%)		
Age (years)				
<44	12 (60.0%)	8 (38.1%)	1.967	0.161
≥44	8 (40.0%)	13 (61.9%)		
Ki67 (%)				
<3	15 (75.0%)	15 (71.4%)	0.067	0.796
≥3	5 (25.0%)	6 (28.6%)		
Tumor volume (cm^3^)				
<3	9 (45.0%)	11 (52.4%)	0.223	0.636
≥3	11 (55.0%)	10 (47.6%)		
Surgical extent				
Residual	3 (15.0%)	7 (33.3%)	2.012	0.156
Gross total	17 (85.0%)	14 (66.7%)		
Invasiveness				
No	3 (14.3%)	13 (65.0%)	11.072	*0.001*
Yes	18 (85.7%)	7 (35.0%)		

## Data Availability

The datasets used and analyzed in the current study are available on the GEO database under the accession number GSE 191113 (https://www.ncbi.nlm.nih.gov/geo/query/acc.cgi?acc=GSE191113).

## Abbreviations

lncRNAs: Long noncoding RNAs
PAs: Pituitary adenomas
NIPA: Noninvasive pituitary adenoma
IPA: Invasive pituitary adenoma
GO: Gene Ontology
KEGG: Kyoto encyclopedia of genes and genomes
qRT-PCR: Real-time quantitative reverse transcription polymerase chain reaction
ROC: Receiver operating characteristic
AUC: Area under the curve
MRI: Magnetic resonance imaging
TR: Transverse
AP: Anteroposterior
CC: Craniocaudal
FDR: False discovery rate
SPSS: Statistical product and service solutions
BP: Biological process
MF: Molecular function
CC: Cellular component.
